# The New Antipyretic

**Published:** 1894-03

**Authors:** 


### THE NEW ANTIPYRETIC.

Every now and then some "discovery" is announced which, for a time, creates a stir in professional circles, and which, as a general thing, soon turns out to be a false alarm and is dropped, to be heard of no more. It were useless to specify instances; numerous cases will be readily recalled. New remedies follow so fast on each other's heels that it would require an extraordinary memory to even recall the names—and most of them have very hard names. For each new remedy brought out preposterous claims are made, and—we have never known it to fail in a single instance—there are men, physicians, who are ready to back up those claims with their experiences, and they hasten to go on record. In a little while the experience of others entirely negatives the results first obtained, or alleged to be obtained, and the remedy is swallowed up in oblivion. Of the thousands of new remedies, some few stand the test of experience and pass into the great materia medica and become standard.

We really believe that were some learned professor, especially a foreigner, with an unpronouncable name, to claim, and publish it in a German journal, that he had observed the most pronounced effects from the administration of small doses of Limburger cheese, there would be a number of American doctors who would swear to it; and for a brief period certain publications would swarm with clinical reports of "Limburger cheese in" such and such disease. The claim has recently been made—somewhere—and on alleged good authority, that small-pox can be cured by light through red glass. Our readers will recall the blue glass craze some years ago. We remember a few years ago a claim was made, and found followers, that carbonate of magnesia, internally administered, would cure warts,

While we are on the subject of the gullibility of the average doctor, we may as well recall the absurd statement that went the rounds of the medical press some time ago, without contradiction, to the effect that a doctor had extracted, whole, a lot of lead which had, in a molten state, gotten into a person's ear. It was claimed that the lead filled the meatus and the eustachian tube, and hardened, making a perfect cast of the internal and middle ear, when this doctor extracted it, and claimed that the specimen was on exhibition, a remarkable arborescent leaden curiosity. There was nothing said of the patient, nor when his funeral took place; on the contrary, the inference was that he suffered no more than if so much sweet oil had entered his ear.

Now and then, however, as stated, a remedy comes to stay. The materia medica has been enriched, in almost every department of therapeutics, with new remedies, and principally of the "coal-tar series;" especially the antipyretics have been derived from that source.

The latest candidate for trial in the class of antipyretics is guaiacol, a derivative of wood-tar, and which can be made by synthesis. Already extravagant claims have been made for it, and the testimony of those who have tried it would indicate that there are strong grounds upon which the claim rests. This remedy, unlike its predecessors of the coal-tar series, is, however, an external remedy. It exerts its antipyretic power in fever, when applied to the surface of the body.

Interesting data on the subject of guaiacol will be found elsewhere in this issue.

As all remedies of this class act by depressing the nervous system, they are all dangerous. We would caution our readers against a too ready acceptance of such a remedy on faith, or until, in the great clinics of the world, its value has been established, and all its properties fully demonstrated. Our physicians, especially the younger ones, ambitious to be thought progressive, and "up to date young men," are apt to be indiscreet in prescribing remedies for which so much has been claimed, and they are liable to kill somebody.

This is especially apt to be the case with typhoid fever, or with that continued form of fever we have in Texas over which there is such a diversity of opinion; for, if there is any fact in medicine now fully established, it is that typhoid fever *can not be aborted;* it must run its course, and the judicious physician will watch the symptoms, prevent complications, and, virtually, *do nothing*. It is a disease, as Stone said of yellow fever, to be "nursed, not treated."

Referring again to typhoid fever, we will venture that the late Professor Fenner, of New Orleans, is responsible for more deaths from that disease than any other one person living or dead. It was a hobby with him that typhoid fever can be aborted with quinine; and he taught it. Older physicians will remember how vehemently he insisted on it, and being recognized thirty years ago as an authority on Southern diseases, he had many disciples. It took his pupils a great many years, and it cost them much bitter experience, to learn that the good doctor, however honestly he believed it, was mistaken. It is the testimony now, of leading physicians in the South, that large doses of quinine are absolutely poisonous, *deadly* to typhoid fever.

